# Proceedings of the Macon Convention

**Published:** 1871-06

**Authors:** 


### *Proceedings of the Macon Convention.*

The following report of the proceedings of the Macon Convention, by Dr. J. N. S., as it appeared in the Daily Constitution of this city, we give to our readers as a true record of what occurred in Macon :

MACON, GEORGIA, July 5, 1871.

The State Medical Convention assembled at 10 o'clock A. M. to-day, at the City Hall; a large number was in attendance. Prayer was offered by the Rev. Dr. Thorn, of Macon, and the welcome address was pronounced by Dr. W. R. Burgess, of this city.

A temporary organization was effected by placing Dr. E. F. Knott, of Griffin, in the chair, who read the call of one hundred and fourteen physicians for the Convention.

On motion of Dr. R. D. Arnold, of Savannah, the members were required to register their names, coupled with the names of the institutions at which they graduated, and the dates of their graduation.

Before entering upon permanent organization, Dr. W. A. Love, of Atlanta, stated that the physicians from the up country did not anticipate that organization would be perfected before meridian — that a large delegation would arrive by the 11 o'clock train.

Dr. R. D. Arnold remarked that notice was given in the call of the day of meeting, and that if gentlemen were not here in time to participate it was their misfortune.

Dr. Love replied that as an omission had occurred in the failure to designate the hour at which the Convention would assemble, that considerations of courtesy should induce us to defer action in this important matter until the gentlemen expected should arrive in the city, *especially* in view of the time of the arrival of the train. It was to be hoped that permanent organization would be postponed until after the hour of eleven.

On motion, however, of Dr. Arnold, the organization was commenced and perfected.

A committee was appointed on nominations, who reported the following names for the respective offices designated:

Dr. S. P. Hawkins, of Americus, President; Dr. F. Walker, of Monticello, First Vice President; Dr. C. H. Hall, of Macon, Second Vice President; W. Duncan, of Savannah, Secretary.

*The Committee on Credentials* consisted of the following: R. D. Arnold, F. Walker, and R. H. Wright, of Macon.

*Committee on Resolutions*—R. D. Arnold, E. D. Alfriend, W. A. Green, J. B. Hinkle, and C. B. Nottingham.

At this stage the up-country delegation arrived, and proceeded to register their names, which register was referred to the Committee on Credentials.

At this juncture the Committee on Resolutions brought and submitted their report, the reading of which was deferred until the Committee on Credentials reported.

Dr. W. A. Love, of Atlanta, obtained the floor, and stated that he and his friends had labored under some difficulties in answering the call of the profession to attend the Convention. That he spoke the sentiments of both himself and his friends in the matter, when he stated that they had responded to this call with the hope of conciliating the difficulties existing between contending elements in the profession, and settling all difficulties amongst its members. With such a hope they had entered this Convention, and to such an end they expected to labor in a spirit of conciliation and concession, if need be, for the sake of peace and harmony.

But while they entered this meeting with such feelings and intentions, they desired to state, distinctly, in registering by invitation that they protested against the authority of this Convention to dictate a course of policy to the State Medical Association. He furthermore desired to state, that he did not understand the language of the "call"—Whereby all questions pertaining to the Atlanta Medical College shall be definitely settled." He stated that all difficulties supposed to exist, so far as the College and its present faculty, trustees, management, etc., were concerned, had been settled many years since, and so acknowledged by the State and the American Medical Associations. He would be pleased to have any gentleman present to state definitely what these difficulties were. In conclusion, in entering the Convention with the status stated, he and his friends hoped and would labor for peace and harmony.

The report of the Credential Committee was received and adopted, and the report of the Committee on Resolutions was read. The preamble and resolutions embraced in their scope a period extending back to the date of granting the original charter of the Atlanta Medical College, its amendment by an act of the Legislature, in 1858, by which such power was transferred from the Trustees to the Faculty, as invested them with authority to remove incumbents from their respective chairs, or to fill the same as they might deem advisable for the interest of the institution. The resolutions also contemplated the various acts, resolutions and protests of the Faculty of the Atlanta Medical College, and also the action of the Georgia Medical Association in 1868, 1869, 1870, and 1871 in relation to the Atlanta Medical College and the Faculty of the same. The report closed with a series of resolutions, the provisions of which had for their object the condemnation of the action taken at the State Medical Association at Americus, which restored to their seats, in said Association, the members of the Faculty of the Atlanta Medical College, who, in the previous year, in Macon, had been expelled from that body.

Dr. R. A. T. Ridley, of LaGrange, was opposed to the resolutions; he announced that the majority should rule, that the action of the majority was respected at the sessions of the State Medical Association in Augusta, Savannah, and Macon, when such measures were adopted as affected the interests of the Atlanta

Medical College, and finally culminated in the expulsion of the members of its former Faculty. As this action was reversed at a subsequent annual session at Americus by a two-thirds majority vote, he was opposed to disturbing the action of the same by convention whose authority to interfere in any event with the proceedings of the State Association he denied. He did not come in the interests of the Savannah Medical College, neither did he come in the interests of the Augusta and Atlanta Medical Schools, but to promote peace; that the seeds of discord had been sown, broadcast. He wished the matter settled now, never to be resuscitated. The breach was made at the session of the Association in Augusta, in 1868, and every subsequent meeting has served to widen it. He devoutly desired that all differences be healed, and that the members of a profession which he had always loved, should be once more restored to permanent, amicable and pleasant relations.

Quite a number participated in the discussion of the resolution. Among them Dr. J. F. Alexander, of Atlanta, and Dr. G. M. McDowell, most of whom protested against any action that proposed to disturb the proceedings of the State Association.

A motion was made by Dr. Ridley to lay the report of the Committee on Resolutions on the table. Hecalled the previous question.

Whilst the President was hesitating to submit the question, Dr. W. A. Green moved to adjourn until 10 o'clock A. M., tomorrow, which was amended by Dr. E. S. Ray, of Atlanta, to adjourn *sine die*.

Dr. E. J. Kirsey, of Columbus, also amended by a proposition to adjourn to a time two days subsequent to the adjournment of the State Medical Association to assemble in annual session in Columbus in April next, which motion prevailed, and the Convention accordingly adjourned.

